# Safety Profile of Epidermal Growth Factor Receptor Tyrosine Kinase Inhibitors: A Disproportionality Analysis of FDA Adverse Event Reporting System

**DOI:** 10.1038/s41598-020-61571-5

**Published:** 2020-03-16

**Authors:** Jing Huang, Long Meng, Bing Yang, Shusen Sun, Zhigang Luo, Hong Chen

**Affiliations:** 1 Department of Respiratory and Critical Care Medicine, The First Affiliated Hospital of Chongqing Medical University, Chongqing, 400016 China; 2 Department of Pharmacy, The First Affiliated Hospital of Chongqing Medical University, Chongqing, 400016 China; 3 Department of Nursing, Chongqing Medical University, Chongqing, 400016 China; 4 Department of Pharmacy Practice, College of Pharmacy and Health Sciences, Western New England University, 1215 Wilbraham Road, Springfield, MA 01119 USA; 5 Department of Pharmacy, Xiangya Hospital Central South University, Changsha, 410008 Hunan China; 6 Institute for Rational and Safe Medication Practices, National Clinical Research Center for Geriatric Disorders, Xiangya Hospital, Central South University, Changsha, 410008 Hunan China; 7 Department of Pharmacy, Shihezi People’s Hospital, Shihezi, 832000 China

**Keywords:** Epidemiology, Non-small-cell lung cancer

## Abstract

Adverse event reports submitted to the US Food and Drug Administration (FDA) were analyzed to map the safety profile of epidermal growth factor receptor-tyrosine kinase inhibitors (EGFR-TKIs). We conducted a disproportionality analysis of the adverse events (AEs) of EGFR-TKIs (gefitinib, erlotinib, afatinib, osimertinib) by data mining using the FDA adverse event reporting system (AERS) database, and by calculating the reporting odds ratios (ROR) with 95% confidence intervals. The FDA AERS database contained 27,123 EGFR-TKI-associated AERs within the reporting period from January 1, 2004 to March 31, 2018. Thirty-three preferred terms (PTs) were selected for analysis, and significant RORs were most commonly observed in the skin, nail, gastrointestinal tract, hepatic, eyes, and lungs. Unexpected adverse drug reactions were found in the “intestinal obstruction” and “hypokalaemia” in gefitinib and erlotinib, “hyponatraemia” in gefitinib, erlotinib and afatinib, “alopecia”in erlotinib, “hair growth abnormal” in afatinib, but not in “nausea” and “vomiting” listed on drug labels. The results of this study are consistent with clinical observation, suggesting the usefulness of pharmacovigilance research should be corroborated with the real-world FAERS data.

## Introduction

Lung cancer is the leading cause of cancer deaths and contributes to over one million deaths worldwide annually^1^. More than 80% of patients with lung cancer are diagnosed as having non-small cell lung cancer (NSCLC), and more than 50% of patients with NSCLC are at an advanced stage when diagnosed^2^. For NSCLC patients who cannot undergo surgery due to an advanced disease stage, platinum-based chemotherapy is the standard of treatment^3^. However, the prognosis of advanced NSCLC remains unsatisfactory due to various chemotherapy-related adverse events (AEs) and increased tumor resistance^4^.

During the last decade, molecularly targeted drugs have increased the effectiveness of NSCLC therapy. Many studies have shown that targeted therapies can significantly improve survival and enhance the quality of life in NSCLC patients^5,6^. The epidermal growth factor receptor (EGFR) as a member of the Her/ErbB receptor family, a principal and potent oncogenic driver in NSCLC, is a therapeutic target. EGFR tyrosine kinase inhibitors (EGFR-TKIs) have higher anti-tumor activities in NSCLC patients who harbor an activating EGFR mutation. With EGFR-TKIs (gefitinib, erlotinib, and afatinib) as first-line treatment for patients carrying sensitizing EGFR mutations with an advanced NSCLC stage, a higher progression-free survival, overall response rate and improved quality of life can be achieved. Osimertinib, which showed a significant objective response rate in EGFR T790M-positive NSCLC, also had been recommended as the first-line treatment^7,8^. These drugs are generally well-tolerated as they have a favorable toxicity profile compared to traditional chemotherapy regimens.

Nevertheless, EGFR-TKIs can still lead to severe AEs such as cutaneous reactions, paronychia, and diarrhea^9^. EGFR-TKI-associated fatal events have also been reported, and they are mainly related to liver or lung toxicities^10,11^. Gefitinib and erlotinib are reversible EGFR- or EGFR/HER2-selective TKI inhibitors, while afatinib is an irreversible EGFR–TKI with a higher affinity for the EGFR kinase domain, possessing more persistent inhibition of EGFR signaling^12^. Osimertinib, as the third irreversible EGFR-TKI, produces beneficial effects through binding to certain mutant forms of EGFR (exon 19 deletion, L858R, and T790M)^13^. Gefitinib and erlotinib share some structural similarities; however, they differ in the pharmacokinetics and substituents attached to the quinazoline and anilino rings, exhibiting different safety profiles^14,15^. The LUXLUNG 3 study showed the incidence and severity of AEs of afatinib were higher compared with the first generation EGFR–TKI. Osimertinib presents a lower rate of ≥1 grade of rash and a lower serious AEs rate in comparsion with gefitinib and erlotinib^16^.The published clinical trials that directly compared the safety of the four agents are extremely rare^17,18^. Differences in safety among these four EGFR-TKIs may have an impact on treatment decisions.

In the past few years, the safety assessment that reflects drug utilization in clinical practice has been conducted by data mining of adverse event spontaneous reporting system (SRS)^19^. The FDA has developed the FDA adverse event reporting system (FAERS), one of the best-known SRSs in the world. Data in the FAERS database are publically available online and are updated quarterly since 2004. Pharmacists, physicians, manufacturers, and other members within and outside the US make spontaneous submissions to the FAERS database. Data mining algorithms, as essential tools in pharmacovigilance, are routinely used for the quantitative detection of signals, i.e., drug-associated AEs^20,21^.

Numerous AE reports have been submitted to the FAERS on EGFR-TKIs. We aimed to assess the reported AEs of EGFR-TKIs through data mining of the FAERS to map the safety profile of EGFR-TKIs.

## Results

From January 1, 2004 to March 31, 2018, the FAERS database received a total of 6,106,629 AE reports, with 4,582 for gefitinib (0.08%), 19,432 for erlotinib (0.32%), 1,540 for afatinib (0.03%), and 1,569 for osimertinib (0.03%). The majority of reports were from USA and Japan. Patients aged >45 years old were preponderance and females contributed a higher overall proportion of AE reports. Most of reports were serious (>60%). A peak in reporting of death was noted for erlotinib (38.9%). The characteristics of AE reports submitted for EGFR-TKIs are described in Table 1.

**Table 1:** Characteristics of reports associated with epidermal growth factor receptor-tyrosine kinase inhibitors (EGFR-TKIs) from January 2004 to March 2018.

	Gefitinib(%)	Erlotinib(%)	Afatinib(%)	Osimertinib(%)
Number of events	4582	19432	1540	1569
Gender				
Female	2370 (51.7)	9481 (48.8)	806 (52.3)	952 (60.7)
Male	1843 (40.2)	8243 (42.4)	486 (31.6)	445 (28.3)
Unknown	369 (8.1)	1708 (8.8)	248 (16.1)	172 (11.0)
Age (year)				
<18	21 (0.5)	19 (0.1)	2 (0.1)	0 (0)
18–44	156 (3.4)	342 (1.8)	46 (3.0)	30 (1.9)
45–64	1149 (25.1)	2866 (14.7)	293 (19.0)	302 (19.2)
65–74	1020 (22.3)	2411 (12.4)	282 (18.3)	308 (19.6)
≥75	848 (18.5)	2028 (10.4)	192 (12.5)	264 (16.8)
Unknown	1388 (30.3)	11766 (60.5)	725 (47.1)	665 (42.4)
Serious outcomes				
Hospitalization	1618 (35.3)	4230 (21.8)	471 (30.6)	499 (31.8)
Disability	205 (4.5)	151 (0.8)	18 (1.2)	32 (2.0)
Life-threatening	344 (7.5)	329 (1.7)	53 (3.4)	60 (3.8)
Death	983 (21.5)	7567 (38.9)	371 (24.1)	566 (36.1)
Reporter country				
USA	1339 (29.2)	11359 (58.5)	346 (22.5)	628 (40.0)
Japan	1090 (23.8)	454 (2.3)	349 (22.7)	416 (26.5)
Other countries	2153 (47.0)	7619 (39.2)	845 (54.9)	525 (33.5)

The 27,123 EGFR-TKI AE reports corresponded with 72,856 drug-reaction pairs, and the drug-pair distribution based on SOCs is shown in Table 2. The most frequently reported SOCs were “General disorders and administration site conditions”, “Skin and subcutaneous tissue disorders” and “Gastrointestinal disorders”. Disproportionality analysis was applied to EGFR-TKIs, both as a drug class and as a single agent. The top 50 list of PTs associated with the most frequently statistically-significant RORs for EGFR-TKIs as a class are shown in the Supplementary Material Table 2.

**Table 2:** Distribution of drug-reaction pairs attributed to EGFR-TKIs according to relevant System Organ Class (SOCs)*.

	Gefitinib	(%)	Erlotinib	(%)	Afatinib	(%)	Osimertinib	(%)
Blood and lymphatic system disorders	63	1.4%	1113	2.1%	340	2.7%	94	2.7%
Cardiac disorders	40	0.9%	559	1.1%	189	1.5%	90	2.5%
Congenital, familial and genetic disorders	15	0.3%	32	0.1%	11	0.1%	3	0.1%
Ear and labyrinth disorders	9	0.2%	162	0.3%	34	0.3%	4	0.1%
Endocrine disorders	2	0.0%	58	0.1%	21	0.2%	18	0.5%
Eye disorders	31	0.7%	892	1.7%	138	1.1%	37	1.0%
Gastrointestinal disorders	1027	23.0%	7639	14.6%	1733	13.7%	429	12.1%
General disorders and administration site conditions	662	14.8%	13229	25.4%	2051	16.2%	885	25.0%
Hepatobiliary disorders	60	1.4%	668	1.3%	557	4.4%	104	3.0%
Immune system disorders	30	0.7%	314	0.6%	66	0.5%	30	0.8%
Infections and infestations	90	2.0%	962	1.8%	349	2.8%	62	1.7%
Injury, poisoning and procedural complications	110	2.5%	1763	3.4%	223	1.8%	98	2.8%
Investigations	91	2.0%	1866	3.6%	789	6.2%	181	5.1%
Metabolism and nutrition disorders	218	4.9%	1956	3.7%	476	3.8%	130	3.7%
Musculoskeletal and connective tissue disorders	61	1.4%	946	1.8%	224	1.8%	86	2.4%
Neoplasms benign, malignant and unspecified (incl cysts and polyps)	586	13.1%	2717	5.2%	817	6.4%	270	7.6%
Nervous system disorders	173	3.9%	1955	3.7%	598	4.7%	127	3.6%
Pregnancy, puerperium and perinatal conditions	0	0.0%	11	0.0%	7	0.1%	0	0.0%
Product issues	2	0.0%	56	0.1%	4	0.0%	1	0.0%
Psychiatric disorders	43	1.0%	534	1.0%	134	1.1%	21	0.6%
Renal and urinary disorders	58	1.3%	438	0.8%	269	2.1%	40	1.1%
Reproductive system and breast disorders	10	0.2%	108	0.2%	43	0.3%	5	0.1%
Respiratory, thoracic and mediastinal disorders	341	7.6%	4504	8.6%	1915	15.1%	410	11.6%
Skin and subcutaneous tissue disorders	675	15.1%	8463	16.2%	1424	11.2%	339	9.6%
Social circumstances	2	0.0%	67	0.1%	31	0.2%	2	0.1%
Surgical and medical procedures	30	0.7%	394	0.8%	72	0.6%	15	0.4%
Vascular disorders	32	0.7%	780	1.5%	159	1.3%	54	1.5%
Total	4463	100.0%	52186	100.0%	12673	100.0%	3534	100.0%

*SOCs are presented in alphabetical order.

In the systematic literature evaluation of the safety profiles of EGFR-TKIs, we identified 9 studies (Supplementary Table 3). The skin, nail, liver, and gastrointestinal and respiratory tracts were the most frequently investigated organ system toxicities. The combined 33 PTs related to EGFR-TKIs were further explored for each individual agent. We found statistically-significant AE RORs for eight organs/systems, including skin, nail, gastrointestinal, hepatobiliary, eye, lung, metabolism, and hair (Table 3). Eighty-eight significant RORs were detected for the four EGFR-TKIs. Among them, 79 AEs were already presented on the drug labels. However, unexpected adverse drug reactions (not listed on drug labels) were uncovered: “intestinal obstruction” and “hypokalaemia” for gefitinib and erlotinib, “hyponatraemia” for gefitinib, erlotinib and afatinib, “alopecia” for erlotinib, and “hair growth abnormal” for afatinib. Both nausea and vomiting, which listed on the drug labels, did not satisfy the criteria for significant RORs.

**Table 3:** Signal strength for epidermal growth factor receptor-tyrosine kinase inhibitors(EGFR-TKIs) at the PT level in FAERS.

	PTs	Gefitinib			Erlotinib			Afatinib			Osimertinib		
		Label^†^	N	ROR (95% CI)	Label^†^	N	ROR (95% CI)	Label^†^	N	ROR (95% CI)	La Lable^†^	N	ROR (95% CI)
Skin	Rash	✓	288	3.82 (3.39,4.31)*	✓	1655	5.36 (5.10,5.64)*	✓	165	6.83 (5.81,8.03)*	✓	62	2.34 (1.82,3.02)*
	Pruritus	✓	61	1.04 (0.81,1.34)	✓	424	1.73 (1.57,1.90)	✓	24	1.22 (0.82,1.83)	✓	18	0.90 (0.56,1.43)
	Acne	✓	52	7.74 (5.88,10.18)*	✓	362	13.22 (11.89,14.70)*	✓	32	14.28 (10.06,20.29)*	✓	4	1.72(0.64,4.57)
	Dermatitis acneiform	✓	41	36.29 (26.57,49.55)*	✓	248	60.01 (52.36,68.79)*	✓	21	54.86 (35.56,84.64)*	✓	15	38.16 (22.89,63.60)*
	Skin disorder	✓	39	6.28 (4.58,8.62)*	✓	87	3.30 (2.67,4.08)*	✓	30	14.54 (10.12,20.88)*	✓	7	3.27 (1.56,6.87)*
	Dehydration	✓	36	7.06 (5.06,9.76)*	✓	100	4.62 (3.79,5.63)*	✓	34	20.03 (14.25,28.17)*	✓	2	1.13 (0.28,4.51)
	Skin ulcer	✓	35	6.55 (4.69,9.14)*	✓	94	4.16 (3.39,5.10)*	✓	5	2.76 (1.15,6.65)*	✓	2	1.08 (0.27,4.33)
	Rash pruritic	✓	20	2.05 (1.32,3.19)*	✓	99	2.41 (1.97,2.93)*	✓	4	1.22 (0.46,3.25)	✓	7	2.10 (1.00,4.41)*
	Skin exfoliation	✓	19	2.15 (1.37,3.37)*	✓	184	4.99 (4.31,5.78)*	✓	12	4.05 (2.30,7.15)*	✓	4	1.32 (0.49,3.52)
	Rash pustular	✓	14	7.36 (4.35,12.45)*	✓	81	10.29 (8.25,12.85)*	✓	7	10.94 (5.20,23.00)*	✓	3	4.58 (1.47,14.23)*
	Skin fissures	✓	8	4.44 (2.21,8.89)*	✓	102	13.89 (11.39,16.95)*	✓	8	13.25 (6.61,26.58)*	✓	5	8.10 (3.36,19.51)*
Nail	Paronychia	✓	37	51.58 (37.09,71.71)*	✓	77	26.23 (20.78,33.11)*	✓	85	389.73 (310.24,489.59)*	✓	17	68.02 (42.00,110.15)*
	Nail disorder	✓	25	16.02 (10.79,23.79)*	✓	59	9.02 (6.96,11.69)*	✓	11	20.88 (11.52,37.84)*	✓	10	18.61 (9.98,34.70)*
Gastrointestina	Diarrhoea	✓	443	4.25 (3.85,4.69)*	✓	2430	5.74 (5.50,5.99)*	✓	447	16.25 (14.55,18.14)*	✓	130	3.58 (2.99,4.28)*
	Nausea	✓	181	1.07 (0.92,1.24)	✓	886	1.24 (1.16,1.33)	✓	67	1.18 (0.92,1.51)	✓	56	0.96 (0.74,1.25)
	Vomiting	✓	171	1.70 (1.46,1.98)	✓	583	1.35 (1.25,1.47)	✓	58	1.71 (1.32,2.23)	✓	40	1.14 (0.84,1.57)
	Decreased appetite	✓	144	3.17 (2.68,3.74)*	✓	649	3.39 (3.14,3.67)*	✓	71	4.72 (3.72,5.99)*	✓	66	4.29 (3.35,5.48)*
	Constipation	✗	38	1.01 (0.73,1.39)	✗	208	1.31 (1.14,1.50)	✗	8	0.63 (0.31,1.26)	✓	18	1.40 (0.88,2.23)
	Stomatitis	✓	34	3.26 (2.33,4.57)*	✓	264	6.09 (5.39,6.88)*	✓	110	33.73 (27.77,40.98)*	✓	22	6.20 (4.07,9.44)*
	Intestinal obstruction	✗	21	2.93 (1.91,4.50)*	✗	72	2.38 (1.88,3.00)*	✗	2	0.83 (0.21,3.31)	✗	1	0.41 (0.06,2.88)
Hepatobiliary	Hepatic function abnormal	✓	161	21.88 (18.67,25.64)*	✓	46	1.41 (1.05,1.88)	✓	5	1.93 (0.80,4.64)	✓	26	10.00 (6.78,14.73)*
	Liver disorder	✓	126	12.04 (10.08,14.38)*	✓	42	0.92 (0.68,1.24)	✓	4	1.10 (0.41,2.94)	✓	35	9.66 (6.91,13.51)*
	Alanine Aminotransferase Increased	✓	94	5.78 (4.71,7.09)*	✓	77	1.09 (0.87,1.37)	✓	3	0.54 (0.17,1.66)	✓	16	2.83 (1.73,4.64)*
	Aspartate Aminotransferase Increased	✓	79	5.46 (4.37,6.82)*	✓	82	1.32 (1.06,1.63)	✓	1	0.20 (0.03,1.44)	✓	11	2.19 (1.21,3.96)*
	Blood bilirubin increased	✓	16	2.11 (1.29,3.45)*	✓	98	3.07 (2.52,3.75)*	✓	1	0.39 (0.06,2.78)	✓	2	0.77 (0.19,3.08)
Eye	Dry eye	✓	11	1.79 (0.99,3.23)	✓	94	3.64 (2.97,4.46)*	✓	4	1.93 (0.72,5.16)	✗	4	1.90 (0.71,5.06)
	Eye irritation	✓	6	0.81 (0.36,1.79)	✓	94	3.00 (2.45,3.68)*	✗	0	-	✗	2	0.78 (0.20,3.14)
Lung	Interstitial lung disease	✓	253	29.18 (25.67,33.16)*	✓	177	4.55 (3.92,5.28)*	✓	28	9.08 (6.25,13.21)*	✓	56	18.20 (13.93,23.77)*
	Pneumonitis	✓	63	14.83 (11.55,19.04)*	✓	93	5.13 (4.18,6.30)*	✓	17	11.78 (7.30,19.02)*	✓	23	15.72 (10.41,23.75)*
Metabolism	Hyponatraemia	✗	28	2.63 (1.81,3.81)*	✗	125	2.78 (2.33,3.32)*	✗	10	2.78 (2.33,3.32)*	✓	14	3.85 (2.27,6.51)*
	Hypokalaemia	✗	22	2.51 (1.65,3.81)*	✗	97	2.62 (2.14,3.20)*	✓	18	6.15 (3.86,9.79)*	✓	5	1.66 (0.69,3.99)
Hair	Alopecia	✓	57	1.62 (1.25,2.11)	✗	347	2.35 (2.11,2.61)*	✗	22	1.86 (1.22,2.84)	✗	2	0.16 (0.04,0.66)
	Hair growth abnormal	✓	12	7.67 (4.34,13.53)*	✓	83	12.94 (10.38,16.12)*	✗	5	9.48 (3.94,22.84)*	✗	1	1.85 (0.26,13.17)

N: the number of adverse events reports.

ROR: the reporting odds ratio.

CI: the confidence interval.

PTs: Preferred Terms.

*Signal detected, see “Methods” for the criteria of detection.

^†^Whether adverse events are mentioned in the drug label or not.

The RORs (95% CI) of the SMQ analysis are summarized in Table 4. The following six SMQs emerged with statistical significant RORs for the four agents: “drug reaction with eosinophilia and systemic symptoms syndrome”, “pseudomembranous colitis”, “interstitial lung disease”, “noninfectious diarrhea”, “severe cutaneous adverse reactions” and “hyponatraemia/SIADH”. The ROR values of the “hepatic disorders” were significant for gefitinib and osimertinib.

**Table 4:** Signal strength for epidermal growth factor receptor-tyrosine kinase inhibitors(EGFR-TKIs) at the SMQ level in FAERS.

SMQs	Gefitinib		Erlotinib		Afatinib		Osimertinib	
	N	ROR (95% CI)	N	ROR (95% CI)	N	ROR (95% CI)	N	ROR (95% CI)
Drug reaction with eosinophilia and systemic symptoms syndrome	1969	3.41 (3.22, 3.62)*	6510	2.29 (2.22, 2.35)*	535	2.41 (2.17, 2.67)*	503	2.13 (1.92, 2.37)*
Hypersensitivity	1006	2.21 (2.06, 2.37)*	5032	2.76 (2.67, 2.85)*	397	2.73 (2.43, 3.06)*	265	1.60 (1.40, 1.82)
Anaphylactic reaction	874	1.66 (1.54, 1.79)	4924	2.40 (2.32, 2.48)*	277	1.55 (1.36, 1.76)	194	0.99 (0.86, 1.16)
Gastrointestinal nonspecific Inflammation and dysfunctional conditions	745	1.49 (1.38, 1.62)	3906	1.94 (1.87, 2.01)	519	3.91 (3.52, 4.35)*	223	1.27 (1.11, 1.47)
Eosinophilic pneumonia	640	4.13 (3.80, 4.49)*	974	1.34 (1.26, 1.43)	91	1.59 (1.29, 1.97)	144	2.56 (2.16, 3.04)*
Hepatic disorders	616	4.39 (4.04, 4.78)*	775	1.17 (1.09, 1.26)	49	0.93 (0.70, 1.23)	124	2.42 (2.02, 2.91)*
Drug related hepatic disorders - comprehensive search	614	4.60 (4.22, 5.01)*	771	1.23 (1.14, 1.37)	49	0.97 (0.73, 1.29)	122	2.50 (2.08, 3.01)*
Pseudomembranous colitis	481	3.92 (3.57,4.31)*	2557	5.11 (4.91, 5.33)*	467	14.56 (13.06, 16.23)*	133	3.09 (2.59, 3.69)*
Noninfectious diarrhoea	476	3.55 (3.23, 3.91)*	2535	4.64 (4.45, 4.84)*	459	13.02 (11.67, 14.52)*	136	2.90 (2.44, 3.46)*
Haemorrhages	443	1.46 (1.33, 1.62)	1318	1.00 (0.94, 1.05)	88	0.83 (0.67, 1.03)	59	0.53 (0.41, 0.69)
Acute central respiratory depression	435	2.14 (1.93, 2.36)*	1115	1.24 (1.17, 1.32)	70	0.97 (0.76, 1.23)	83	1.14 (0.91, 1.42)
Interstitial lung disease	402	13.11 (11.83, 14.53)*	421	3.01 (2.73, 3.32)*	52	4.73 (3.59, 6.24)*	91	8.34 (6.75, 10.31)*
Cardiomyopathy	355	1.03 (0.92, 1.15)	1145	0.77 (0.72, 0.81)	84	0.71 (0.57, 0.88)	135	1.15 (0.97,1.38)
Haematopoietic cytopenias	329	1.90 (1.70, 2.13)	1073	1.44 (1.35, 1.53)	52	0.86 (0.65, 1.13)	114	1.92 (1.59, 2.33)
Gastrointestinal perforation, Ulceration, haemorrhage or obstruction	308	1.78 (1.59, 2.00)	1149	1.56 (1.47, 1.65)	174	3.15 (2.69, 3.69)*	65	1.07 (0.83, 1.37)
Chronic kidney disease	208	1.64 (1.43, 1.89)	523	0.96 (0.88, 1.04)	46	1.06 (0.79, 1.43)	40	0.90 (0.66, 1.24)
Acute renal failure	190	1.76 (1.52, 2.04)	362	0.77 (0.70, 0.86)	62	1.71 (1.32, 2.20)	31	0.82 (0.57, 1.17)
Oropharyngeal disorders	153	1.00 (0.85, 1.17)	670	1.03 (0.95, 1.11)	166	3.49 (2.97, 4.10)*	45	0.85 (0.63, 1.15)
Severe cutaneous adverse reactions	138	5.32 (4.49, 6.30)*	700	6.49 (6.01,7.00)*	147	18.09 (15.25, 21.44)*	54	6.09 (4.64, 7.99)*
Agranulocytosis	106	1.44 (1.19, 1.75)	307	0.98 (0.87, 1.09)	36	1.45 (1.05, 2.02)	19	0.74 (0.47, 1.17)
Biliary disorders	96	1.50 (1.22, 1.84)	333	1.22 (1.10, 1.36)	16	0.74 (0.45, 1.20)	10	0.45 (0.24, 0.84)
Hyponatraemia/SIADH	55	2.73 (2.10, 3.57)*	197	2.31 (2.01, 2.66)*	15	2.21 (1.33, 3.68)*	22	3.20 (2.10, 4.87)*
Corneal disorders	36	1.13 (0.81, 1.56)	301	2.25 (2.01, 2.52)*	11	1.02 (0.57, 1.85)	14	1.28 (0.76, 2.17)
Ocular infections	31	1.81 (1.27, 2.58)	183	2.54 (2.19, 2.94)*	12	2.09 (1.18, 3.69)*	11	1.88 (1.04, 3.40)
Conjunctival disorders	22	1.63 (1.07, 2.47)	168	2.96 (2.54, 3.44)*	12	2.65 (1.50, 4.67)*	10	2.16 (1.16, 4.02)*
Proteinuria	20	2.58 (1.67, 4.01)*	51	1.55 (1.18, 2.04)	3	1.15 (0.37, 3.57)	3	1.13 (0.36, 3.50)

N: the number of adverse events reports.

ROR: the reporting odds ratio.

CI: the confidence interval.

SMQs: standardized MedDRA queries.

*Signal detected, see “Methods” for the criteria of detection.

## Discussion

Whereas some similarities exist, the safety properties of EGFR-TKIs are different. Nevertheless, published clinical trials that have assessed the AEs of different EGFR-TKIs are lacking. To our knowledge, this is the first such safety study from the data mining of the FAERS. In our study, several organs or tissues are found to be involved in toxicities. We find statistically-significant RORs for EGFR-TKIs in the skin, nail, gastrointestinal tract, liver, eyes, and lungs, in contrast, adverse effects in the cardiac, renal, neurological, hematopoietic systems are less common. We uncovered significant disproportionality of novel AEs (intestinal obstruction, hypokalaemia, hyponatraemia) in gefitinib, (intestinal obstruction, hypokalaemia, hyponatraemia, alopecia) in erlotinib, and (hyponatraemia, hair growth abnormal) in afatinib.

As EGFR plays an essential role in epithelial maintenance, EGFR-TKIs may impair the keratinocyte migration, growth, and chemokine expression, leading to inflammatory cell recruitment and cutaneous injury. Skin toxicities are the most common side effects associated with anti-EGFR therapy. There are diverse dermatological symptoms ranging from rash, dermatitis acneiform, mucosal inflammation, skin ulcer, and skin fissures to potentially fatal reactions such as skin exfoliation. Our findings present significant RORs for these four drugs in a rash, rash pustular, dermatitis acneiform, skin fissures, and skin disorder. Preapproval clinical trials reported that the incidences of any grade skin reactions of all types are 90% with afatinib, 85% with erlotinib, 47% with gefitinib, and 34–58% with osimertinib^22^. Clinicians should be aware of the potential toxic skin reactions with EGFR-TKIs. The nail toxicity reported to FAERS with EGFR-TKIs included paronychia, onycholysis, and nail disorder. About 10 and 15% of patients experience paronychia during 4–8 weeks’ EGFR-TKIs treatment^23^. The disproportional RORs of paronychia obtained suggests that these four EGFR-TKIs may increase the risk of nail disorder. In line with several clinical trials, paronychia is more common with afatinib (33%–57%) and osimertinib (~25%) but rarely seen with erlotinib (~4%) and gefitinib (3–14%)^24–26^. Most instances of paronychia related to EGFR-TKI therapy are mild, but in clinical trials of afatinib, treatment-related paronychia led to dose reductions in 14% of patients^27^.

The EGFR signal pathway is implicated in hair cycle regulation and the maintenance of normal hair follicles. EGFR-TKIs result in suppression of the progression from the anagen to the telogen phase, and lead to the dis-organisation of hair follicle and inflammation^28^. Although “alopecia” and “hair growth abnormal” are unexpected AEs in erlotinib and afatinib, the disproportionality is significant in our analysis. When patients are treated with EGFR-TKIs, 0–13% patients manifest hair abnormalities, including alopecia, eyelash changes and excessive hypertrichosis^29^. It has been reported that 1.9–4.9% of patients with erlotinib experienced alopecia^30^. Doxycycline and steroid agents could be considered a potential therapeutic option for EGFR-TKIs-related alopecia^31^.

Gastrointestinal events are frequent during EGFR-TKIs treatment, including stomatitis, nausea, and diarrhea, vomiting, and decreased appetite, of which diarrhea is the most common event. We revealed significant disproportionality of diarrhea at specific SMQs and PT levels in these four EGFR-TKIs. Excess secretion of chloride with EGFR-TKI treatment results in the secretory form of diarrhea^32^. Our study is consistent with other systematic reviews^33^. The incidence of diarrhoea in EGFR-TKIs varied across different trials; with estimated incidences being 65−96% for afatinib, 54–62% for erlotinib, 58% for osimertinib, and 29% for gefitinib^34^. Severe diarrhea can lead to dehydration and electrolyte imbalance including hyponatraemia and hypokalaemia, which are not on the labels of gefitinib and erlotinib. Still a significant disproportionality is detected in our analysis. The non-significant ROR for nausea, vomiting is detected in EGFR-TKIs, while these events are all described on package inserts. Mauricio *et al*. examined 28 studies that revealed the risk of nausea and vomiting was not significant among patients who received gefitinib, afatinib, and erlotinib, which was in agreement with our results^35^. Significant RORs were not observed for nausea and vomiting due to potential underreporting.

We also find a disproportionate association with intestinal obstruction for gefitinib and erlotinib. A 57-years old patient who had no history of the gastrointestinal disease was reported with a diagnosis of intestinal obstruction after using gefitinib^36^. However, there are rare cases reported in gefitinib and erlotinib. The risk of intestinal obstruction in gefitinib and erlotinib remains to be demonstrated with clinical data.

Hepatotoxicity is one of the class-related severe safety issues for EGFR-TKIs in clinical practice. In this study, the ROR values are statistically significant for hepatic disorders in gefitinib and osimertinib but not in afatinib and erlotinib at SMQ and PT levels. Based on a pooled analysis, the occurrence of hepatotoxicity is significantly higher in the gefitinib group than in the erlotinib group [18% vs. 5.4%; OR: 3.71, 95% CI (2.12,6.49); P < 0.0001]^37^. The incidence of grade ≥3 increase in the circulating levels of ALT/AST is higher with gefitinib compared with afatinib (8.2/2.5 vs. 0/0%) in the LUX-Lung7 trial^34^. In a study of 411 patients treated with osimertinib, elevations in liver enzymes are observed in 12.2% (ALT and AST) of the patients^38^. Gefitinib and erlotinib share a similar structure, but they differ in the substituents attached to the quinazoline and anilino rings. The different hepatotoxicity might be caused by the minor differences in the chemical structures of these compounds. From another perspective, CYP3A4 plays a major role in the metabolism of gefitinib and erlotinib, while CYP2D6 provides a significant alternative pathway for the elimination of gefitinib^39^. Decreased CYP2D6 activity might at least partially account for gefitinib-induced hepatotoxicity^40^. It should be noted that CYP3A4 inducers, anti-acid secreting agents, liver metastasis, and age ≥65 are related with EGFR TKI-induced hepatotoxicity^41^.

Interstitial lung disease (ILD) is defined as non-specific symptoms, including fever, cough, dyspnea, and hypoxemia. ILD is considered as the most serious and fatal AEs in EGFR-TKI treatment. EGFR is involved in the progression of repairing lung injury^42^. Therefore, inhibition of EGFR signaling could impair the repair capability of pulmonary cells and then exacerbate pulmonary injury. Other research revealed there a possible cause of allergic reaction to EGFR-TKIs^43^. In our study, a disproportionate association with ILD is suggested for all four EGFR-TKIs, which is strongly in agreement with the findings of other clinical trials^14,44,45^. Patients should be screened at each visit for signs of ILD, which includes cough or low-grade fever and acute onset of dyspnea. Discontinuation of the culprit EGFR-TKI, provision of supportive care including antibacterial agents if appropriate, and administration of steroids have been considered in patients diagnosed of TKI-induced ILD.

Drug reaction with eosinophilia and systemic symptoms (DRESS) is a severe, potentially fatal drug reaction, which is associated with a mortality rate of up to 10%. The clinical features of DRESS are atypical and diverse, including skin eruption, fever, hematologic abnormalities, lymphadenopathy and internal organ damage^46^. The PT terms of manifestations related to DRESS, such as “dermatitis exfoliative”, “rash” and “hepatic enzyme increased” are assoicated with EGFR-TKI agents in our study^47^. This is the reason that the four EGFR-TKIs present statistical significances in SMQ: “drug reaction with eosinophilia and systemic symptoms syndrome”.

Overall, our data point to the fact that the risk profiles of EGFR-TKIs are different, clinicians should consider specific risk factors to select the optimal treatment agent for individual patient. The data mining FAERS database is considered to be a valuable tool; however, causality cannot be established based only on data from FAERS due to the following inherent database limitations: (1) we estimate ROR values based on the reported frequency of drug-event combinations for the studied drug, and values are adjusted based on the rates reported for other drugs and the rates of all other AEs reported for the studied drug. However, despite the limitation with the ROR method, it is still a powerful tool to explore an increased risk of AE reporting. (2) the incomplete data in the AERS are growing (e.g., missing patient demographic information), variable reporting rates overtime, underreporting, duplicate reports, unverified source of submitted data, and inability and missing information^48^. Therefore, causality cannot be confirmed based on the FAERS data alone. However, the following procedures were performed to address the limitations of FAERS disproportionality data analysis including cleaning AE reports before analysis, correcting the disproportionality analysis at the SMQ level for the underestimation of drug-event combinations due to variabilities in the PTs selection to describe the same AE; and applying a stricter signal threshold (ROR ≥ 2.0, the lower bound of the 95% CI > 1.0, N ≥ 3) to enhance the certainty of identifying relevant AEs. (3) incomplete data of AERs, high mortality rate during cancer-therapy and difficulties in retracing the precise sequence of therapy from medical histories do not allow providing rigorous exploration of the onset and fatality of EGFR-TKI-associated AEs.

## Conclusions

The safety profiles of gefitinib, erlotinib, afatinib, and osimertinib, are reviewed using the AEs submitted to the FAERS. Based on the 27,123 reports from 2004 to 2018, AEs with EGFR-TKIs occur in many organs/tissues (skin, nail, gastrointestinal tract, liver, eyes, and lungs). There is a difference in the disproportionality between different EGFR-TKIs-associated adverse events, which has a negative influence on the quality of life and even leads to fatal outcomes. The usefulness of pharmacovigilance research should be corroborated with the real-world FAERS data; however, further clinical trials are required to confirm our findings.

## Methods

### Data sources

Four EGFR-TKIs, erlotinib, gefitinib, afatinib and osimertinib were selected as study drugs. Data from the FAERS database were fully anonymized by regulatory authorities. We extracted relevant data from the public release of the FAERS database for the pharmacovigilance disproportionality analysis, which covered the period from January 1, 2004 to March 31, 2018. OpenVigil FDA, a validated pharmacovigilance tool, is adapted to query FAERS data using the openFDA application programming interface (API) for accessing the FDA drug-event database with the additional openFDA drug mapping and duplicate detection functionality^49,50^, and it is used in many pharmacovigilance studies^51,52^. OpenVigil operates only on the cleaned FDA data by deleting duplicates or reports with missing data^49^. After data cleaning by OpenVigil FDA, 6,106,629 reports from 2004 Q1 to 2018 Q1 remained. Among the drugs and AEs in the reports, we only selected reports with drug codes of “Primary Suspect or “Secondary Suspect”.

### Definition of adverse events

Adverse events in the FAERS database are coded according to the terminology preferred by the Medical Dictionary for Regulatory Activities (MedDRA) Preferred Terms (PTs). The hierarchical structure of MedDRA allows grouping of PTs into relevant System Organ Class (SOC). In addition, different PTs can also be combined to define a medical condition or area of interest through an algorithmic approach known as the Standardized MedDRA Query (SMQ).

Disproportionality analysis was first performed using all existing PTs to identify EGFR-TKI class safety profiles, and key toxicities identified were characterized in terms of PT-level specific signs/symptoms for further disproportionality analysis among the four EGFR-TKIs (as a single drug).

Then for the disproportionality analysis, we also selected the following five characteristic AEs from the overview of EGFR-TKIs safety-related system reviews: pruritus (PT10037087), nausea (PT10028813), vomiting (PT10047700), constipation (PT10010774), and alanine aminotransferase increased (PT10001551) for mining. In the overview of systematic reviews, published articles written in English (from 1/1/1967 to 30/4/2019) reporting the safety of EGFR-TKIs in human patients were identified through computerized literature searches using MEDLINE. The search strategy and inclusion eligibility are provided as an electronic Supplementary Material (Supplementary Tables 3–4). Finally, the safety profile of each of the EGFR-TKIs was examined through SMQ analysis.

Unexpected adverse drug reaction was defined as any significant AE uncovered which was not listed in the FDA drug labelling. To minimize the existence of an “indication bias” (i.e., the indication for which the drug is prescribed is reported as an AE), PTs and SMQs associated with lung cancer-related signs and complications were removed for analysis. Namely, we only analyzed adverse events caused by drugs not by disease state.

### Data mining algorithm

This is a case/non-case study, which can be viewed as a case-control analysis. “Cases” were defined as patients who reported a specific AE, while “non-cases” consisted of patients associated with all other reports. We performed a disproportionality analysis using the reporting odds ratio (ROR) to assess whether there is a signal for a potentially increased risk of drug-associated AE among EGFR-TKIs. When a drug is more likely to induce a specific AE compared to all other drugs, it typically receives a higher ROR score^53^. The ROR is the ratio of the odds of reporting AEs versus all other reactions associated with EGFR-TKIs compared with the reporting odds for all other drugs present in the database. To compare the “cases” and “non-cases,” we calculated the RORs as (a × d)/(b × c). The RORs were expressed as point estimates with a 95% confidence interval (CI) (Supplementary Table 1). The signal was considered positive if the lower limit of 95% CI was >1 and the reported number was >2, and at least three cases were required^48^. All analyses were performed using SPSS (version 23.0) and Microsoft EXCEL 2010.

## Supplementary information

Supplementary information

## References

[1] Torre LA (2015). Global cancer statistics, 2012. Ca A Cancer J. Clinicians.

[2] Vázquez S (2016). EGFR testing and clinical management of advanced NSCLC: a Galician Lung Cancer Group study (GGCP 048-10). Cancer Manag. Res..

[3] Schiller JH (2002). Comparison of Four Chemotherapy Regimens for Advanced Non–Small-Cell Lung Cancer. N. Engl. J. Med..

[4] Molina JR, Yang P, Cassivi SD, Schild SE, Adjei AA (2008). Non-small cell lung cancer: epidemiology, risk factors, treatment, and survivorship. Mayo Clin. Proc..

[5] Mok TS (2011). Gefitinib or Carboplatinï¿½Paclitaxel in Pulmonary Adenocarcinoma. *The*. J. Evidence-Based Med..

[6] Solomon BJ (2014). First-line crizotinib versus chemotherapy in ALK-positive lung cancer. N. Engl. J. Med..

[7] Yang JC (2015). Clinical activity of afatinib in patients with advanced non-small-cell lung cancer harbouring uncommon EGFR mutations: a combined post-hoc analysis of LUX-Lung 2, LUX-Lung 3, and LUX-Lung 6. Lancet Oncol..

[8] Mok TS (2017). Osimertinib or Platinum-Pemetrexed in EGFR T790M-Positive Lung Cancer. N. Engl. J. Med..

[9] Maemondo M (2010). Gefitinib or chemotherapy for non-small-cell lung cancer with mutated EGFR. N. Engl. J. Med..

[10] Ren S (2012). Fatal asymmetric interstitial lung disease after erlotinib for lung cancer. Respiration; Int. Rev. Thorac. Dis..

[11] Schacherkaufmann S, Pless M (2010). Acute Fatal Liver Toxicity under Erlotinib. Case Rep. Oncol..

[12] Modjtahedi H, Cho BC, Michel MC, Solca F (2014). A comprehensive review of the preclinical efficacy profile of the ErbB family blocker afatinib in cancer. Naunyn-Schmiedeberg“s Arch. Pharmacology.

[13] Yi L, Fan J, Qian R, Luo P, Zhang J (2019). Efficacy and safety of osimertinib in treating EGFR-mutated advanced NSCLC: A meta-analysis. Int. J. cancer.

[14] Ding PN (2017). Risk of Treatment-Related Toxicities from EGFR Tyrosine Kinase Inhibitors: A Meta-analysis of Clinical Trials of Gefitinib, Erlotinib, and Afatinib in Advanced EGFR-Mutated Non-Small Cell Lung Cancer. J. Thorac. oncology: Off. Publ. Int. Assoc. Study Lung Cancer.

[15] Togashi Y (2011). Differences in adverse events between 250 mg daily gefitinib and 150 mg daily erlotinib in Japanese patients with non-small cell lung cancer. Lung cancer.

[16] Mezquita L, Varga A, Planchard D (2018). Safety of osimertinib in EGFR-mutated non-small cell lung cancer. Expert. Opin. drug. Saf..

[17] Lin JZ, Ma SK, Wu SX, Yu SH, Li XY (2018). A network meta-analysis of nonsmall-cell lung cancer patients with an activating EGFR mutation: Should osimertinib be the first-line treatment?. Med..

[18] Suh CH (2018). Pneumonitis in advanced non-small-cell lung cancer patients treated with EGFR tyrosine kinase inhibitor: Meta-analysis of 153 cohorts with 15,713 patients: Meta-analysis of incidence and risk factors of EGFR-TKI pneumonitis in NSCLC. Lung cancer.

[19] Dumouchel W (1999). Bayesian Data Mining in Large Frequency Tables, with an Application to the FDA Spontaneous Reporting System. Am. Statistician.

[20] Kadoyama K (2011). Hypersensitivity reactions to anticancer agents: data mining of the public version of the FDA adverse event reporting system, AERS. J. Exp. Clin. cancer research: CR.

[21] Raschi E (2019). Toxicities with Immune Checkpoint Inhibitors: Emerging Priorities From Disproportionality Analysis of the FDA Adverse Event Reporting System. Target. Oncol..

[22] Charles C (2016). Impact of dermatologic adverse events induced by targeted therapies on quality of life. Crit. Rev. Oncology/hematology.

[23] Noushin H, Haley N, Susan B (2008). Chemotherapeutic agents and the skin: An update. J. Am. Acad. Dermatology.

[24] Thatcher N (2005). Gefitinib plus best supportive care in previously treated patients with refractory advanced non-small-cell lung cancer: results from a randomised, placebo-controlled, multicentre study (Iressa Survival Evaluation in Lung Cancer). Lancet.

[25] Rafael R (2012). Erlotinib versus standard chemotherapy as first-line treatment for European patients with advanced EGFR mutation-positive non-small-cell lung cancer (EURTAC): a multicentre, open-label, randomised phase 3 trial. Lancet Oncol..

[26] Sequist LV (2013). Phase III study of afatinib or cisplatin plus pemetrexed in patients with metastatic lung adenocarcinoma with EGFR mutations. J. Clin. Oncol..

[27] Melosky B, Hirsh V (2014). Management of common toxicities in metastatic NSCLC related to anti-lung cancer therapies with EGFR-TKIs. Front. Oncol..

[28] Galimont-Collen AF, Vos LE, Lavrijsen AP, Ouwerkerk J, Gelderblom H (2007). Classification and management of skin, hair, nail and mucosal side-effects of epidermal growth factor receptor (EGFR) inhibitors. Eur. J. cancer.

[29] Peng Y (2019). Update review of skin adverse events during treatment of lung cancer and colorectal carcinoma with epidermal growth receptor factor inhibitors. Biosci. trends.

[30] Scagliotti Giorgio V., Shuster Dale, Orlov Sergey, von Pawel Joachim, Shepherd Frances A., Ross Jeffrey S., Wang Qiang, Schwartz Brian, Akerley Wallace (2018). Tivantinib in Combination with Erlotinib versus Erlotinib Alone for EGFR-Mutant NSCLC: An Exploratory Analysis of the Phase 3 MARQUEE Study. Journal of Thoracic Oncology.

[31] Chen CA, Costa DB, Wu PA (2015). Successful treatment of epidermal growth factor receptor inhibitor-induced alopecia with doxycycline. JAAD case Rep..

[32] Uribe JM, Gelbmann CM, Traynor-Kaplan AE, Barrett KE (1996). Epidermal growth factor inhibits Ca(2+)-dependent Cl- transport in T84 human colonic epithelial cells. Am. J. Physiol..

[33] Zhang Y (2016). Optimized selection of three major EGFR-TKIs in advanced EGFR-positive non-small cell lung cancer: a network metaanalysis. Oncotarget.

[34] Takeda M, Nakagawa K (2017). Toxicity profile of epidermal growth factor receptor tyrosine kinase inhibitors in patients with epidermal growth factor receptor gene mutation-positive lung cancer. Mol. Clin. Oncol..

[35] Burotto M, Manasanch EE, Wilkerson J, Fojo T (2015). Gefitinib and erlotinib in metastatic non-small cell lung cancer: a meta-analysis of toxicity and efficacy of randomized clinical trials. oncologist.

[36] Liang Y-C, Wu G, Cheng J, Yu D-D, Wu H-G (2015). Gefitinib-induced intestinal obstruction in advanced non-small cell lung carcinoma: A case report. Oncol. Lett..

[37] Takeda M, Okamoto I, Nakagawa K (2015). Pooled safety analysis of EGFR-TKI treatment for EGFR mutation-positive non-small cell lung cancer. Lung Cancer.

[38] Shah RR, Shah DR (2019). Safety and Tolerability of Epidermal Growth Factor Receptor (EGFR) Tyrosine Kinase Inhibitors in Oncology. Drug. Saf..

[39] Shah (2013). Hepatotoxicity of Tyrosine Kinase Inhibitors: Clinical and Regulatory;Perspectives. Drug. Saf. An. Int. J. Med. Toxicol. Drug Experience.

[40] Takashi K (2011). Safe and successful treatment with erlotinib after gefitinib-induced hepatotoxicity: difference in metabolism as a possible mechanism. J. Clin. Oncol. Off. J. Am. Soc. Clin. Oncol..

[41] Kim MK (2018). Risk factors for erlotinib-induced hepatotoxicity: a retrospective follow-up study. BMC cancer.

[42] Aoe, K. *et al*. *Sudden onset of interstitial lung disease induced by gefitinib in a lung cancer patient with multiple drug allergy*. **25**, 415–418 (2005). 15816604

[43] Madtes DK, Rubenfeld G, Klima LD, Milberg JA, Clark JG (1998). Elevated Transforming Growth Factor- α Levels in Bronchoalveolar Lavage Fluid of Patients with Acute Respiratory Distress Syndrome. Am. J. Respir. Crit. Care Med..

[44] Hong D, Zhang G, Zhang X, Lian X (2016). Pulmonary Toxicities of Gefitinib in Patients With Advanced Non-Small-Cell Lung Cancer: A Meta-Analysis of Randomized Controlled Trials. Med..

[45] Marinis F (2019). ASTRIS: a global real-world study of osimertinib in>3000 patients with EGFR T790M positive non-small-cell lung cancer. Future Oncol..

[46] Cacoub Patrice, Musette Philippe, Descamps Vincent, Meyer Olivier, Speirs Chris, Finzi Laetitia, Roujeau Jean Claude (2011). The DRESS Syndrome: A Literature Review. The American Journal of Medicine.

[47] MedDRA, MSSO. Introductory Guide for Standardised MedDRA Queries (SMQs) Version 21.0. Available from, https://www.meddra.org/sites/default/files/guidance/file/smq_intguide_21_0_english.pdf.Accessed Dec 26, (2019).

[48] Bate A, Evans SJW (2010). Quantitative signal detection using spontaneous ADR reporting. Pharmacoepidemiol. Drug. Saf..

[49] Böhm R (2016). OpenVigil FDA - Inspection of USAmerican Adverse Drug Events Pharmacovigilance Data and Novel Clinical Applications. PLoS one.

[50] Böhm R, Höcker J, Cascorbi I, Herdegen T (2012). OpenVigil—free eyeballs on AERS pharmacovigilance data. Nat. Biotechnol..

[51] Ji H-H, Tang X-W, Dong Z, Song L, Jia Y-T (2019). Adverse event profiles of anti-CTLA-4 and anti-PD-1 monoclonal antibodies alone or in combination: analysis of spontaneous reports submitted to FAERS. Clin. drug. investigation.

[52] Siafis S, Papazisis G (2018). Detecting a potential safety signal of antidepressants and type 2 diabetes: a pharmacovigilance‐pharmacodynamic study. Br. J. Clin. pharmacology.

[53] Meng L (2019). Assessing fluoroquinolone‐associated aortic aneurysm and dissection: Data mining of the public version of the FDA adverse event reporting system. Int. J. Clin. Pract..

