# Supplementary material for: Safety Profile of Epidermal Growth Factor Receptor Tyrosine Kinase Inhibitors: A Disproportionality Analysis of FDA Adverse Event Reporting System
Source: Sci Rep. 2020 Mar 16;10:4803. doi: 10.1038/s41598-020-61571-5 (PMC7075865; doi:10.1038/s41598-020-61571-5)
Supplement: Supplementary file 1 — Supplementary information [file 41598_2020_61571_MOESM1_ESM.docx]

Safety Profile of Epidermal Growth Factor Receptor Tyrosine Kinase Inhibitors: A Disproportionality Analysis of FDA Adverse Event Reporting System

**Jing Huang^1,+^, Long Meng^2,+^,Bing Yang^3^,Shusen Sun****^4,5,6^, Zhigang Luo^7^, Hong Chen^1^***

^1^Department of Respiratory and Critical Care Medicine, The First Affiliated Hospital of Chongqing Medical University, Chongqing 400016 China,

^2^Department of Pharmacy, The First Affiliated Hospital of Chongqing Medical University, Chongqing 400016 China,

^3^Department of Nursing, Chongqing Medical University, Chongqing 400016 China.

^4^Department of Pharmacy Practice, College of Pharmacy and Health Sciences, Western New England University, 1215 Wilbraham Road, Springfield, MA 01119 USA.

^5^Department of Pharmacy, Xiangya Hospital Central South University, Changsha 410008, Hunan,China.

^6^Institute for Rational and Safe Medication Practices, National Clinical Research Center for Geriatric Disorders, Xiangya Hospital, Central South University, Changsha 410008, Hunan, China.

^7^Department of Pharmacy, Shihezi People's Hospital, Shihezi 832000 China.

* E-mail: hopechen2019@126.com

^+^these author contributed equally to this work

Supplementary table 1 Fourfold table for measure of disproportionality

|  | Adverse event of interest | All other adverse events | Total |
| --- | --- | --- | --- |
| Drug of interest | a | b | a+b |
| All other drugs | c | d | c+d |
| Total | a+c | b+d | a+b+c+d |

ROR ＝(a×d)/(b×c) 95%CI＝e^ln(ROR)±1.96√(1^*^/a+^*^1^*^/b+^*^1^*^/c+^*^1^*^/d)^*

Supplementary table 2 The top 50 list of PTs with most frequently significant ROR for EGFR-TKIs

| Type of adverse events  (preferred terms level) | N | ROR | Lower limit of the 95% two-sided (CI) |
| --- | --- | --- | --- |
| Death | 5759 | 5.98 | (5.8) |
| Rash^*^ | 3639 | 9.19 | (8.87) |
| Diarrhoea^*^ | 3407 | 5.86 | (5.65) |
| Malignant neoplasm progression | 1336 | 17.68 | (16.71) |
| Disease progression | 1007 | 7.86 | (7.38) |
| Decreased appetite^*^ | 920 | 3.49 | (3.27) |
| Pneumonia^*^ | 747 | 2.06 | (1.92) |
| Dehydration | 605 | 3.55 | (3.28) |
| Interstitial lung disease^*^ | 502 | 9.67 | (8.84) |
| Acne^*^ | 446 | 11.86 | (10.77) |
| Stomatitis^*^ | 426 | 7.20 | (6.53) |
| Alopecia^*^ | 425 | 2.08 | (1.89) |
| Pleural effusion | 421 | 5.21 | (4.73) |
| Respiratory failure | 377 | 3.72 | (3.36) |
| Incorrect dose administered | 356 | 2.11 | (1.9) |
| Neoplasm malignant | 356 | 4.24 | (3.81) |
| Dermatitis acneiform^*^ | 321 | 59.37 | (52.48) |
| Metastases to central nervous system | 315 | 25.86 | (23) |
| Lung neoplasm malignant | 291 | 7.81 | (6.95) |
| Lung disorder | 258 | 4.54 | (4.01) |
| Haemoptysis | 244 | 7.13 | (6.27) |
| Hepatic function abnormal^*^ | 234 | 5.30 | (4.65) |
| Epistaxis | 216 | 2.27 | (1.98) |
| Skin exfoliation^*^ | 216 | 4.24 | (3.7) |
| Paronychia^*^ | 209 | 60.25 | (51.71) |
| Liver disorder^*^ | 203 | 3.25 | (2.83) |
| Pneumonitis^*^ | 193 | 7.85 | (6.79) |
| Hyponatraemia^*^ | 174 | 2.80 | (2.41) |
| Aspartate aminotransferase increased^*^ | 173 | 2.02 | (1.74) |
| Mucosal inflammation^*^ | 167 | 5.64 | (4.83) |
| Skin disorder^*^ | 161 | 4.46 | (3.82) |
| Neoplasm progression | 150 | 5.08 | (4.32) |
| Hypoxia | 145 | 3.76 | (3.19) |
| Hypokalaemia^*^ | 142 | 2.78 | (2.35) |
| Pulmonary oedema | 139 | 2.29 | (1.94) |
| Skin ulcer^*^ | 136 | 4.37 | (3.69) |
| Metastases to bone | 135 | 8.54 | (7.18) |
| Rash pruritic | 129 | 2.27 | (1.91) |
| Drug resistance | 123 | 5.76 | (4.81) |
| Non-small cell lung cancer | 123 | 34.90 | (28.86) |
| Skin fissures^*^ | 121 | 12.00 | (9.99) |
| Metastases to liver | 120 | 6.64 | (5.53) |
| Blood bilirubin increased^*^ | 117 | 2.65 | (2.21) |
| Dry eye^*^ | 113 | 3.16 | (2.63) |
| Pneumothorax | 113 | 5.00 | (4.15) |
| Eye irritation^*^ | 102 | 2.35 | (1.94) |
| Nail disorder^*^ | 102 | 11.51 | (9.43) |
| Rash pustular^*^ | 101 | 9.34 | (7.66) |
| Hair growth abnormal^*^ | 99 | 11.23 | (9.17) |
| Intestinal obstruction^*^ | 96 | 2.29 | (1.87) |

N: the number of ad­verse events reports.

ROR: the reporting odds ratio

CI: the confidence interval

^*^included for further analysis

Supplementary table 3 Search strategy of the overview of system reviews

| ***Source*** | MEDLINE - PubMed |
| --- | --- |
| ***Dictionary*** | MESH Term |
| ***Language*** | English |
| ***Period*** | 1/1/1967-30/4/2019 |
| ***Search Terms*** | (gefitinib OR erlotinib OR afatinib OR osimertinib) AND  ("adverse drug reactions" OR "adverse drug reaction" OR safety OR toxicity) |
| ***Restrictions/filters*** | human, English, systematic review, meta-analysis |
| ***Additional procedure undertaken*** | full text assessment |
| ***Final references*** | 9 |

Supplementary table 4 Characteristics of the included studies

| **Author** | **Year** | **Patient/ Population** | **EGFR-TKIs treatment** | **Study design** | **Number of patients** | | **Safty outcome** | |
| --- | --- | --- | --- | --- | --- | --- | --- | --- |
| Zhang[^1^](#_ENREF_1) | 2016 | Advanced EGFR-positive NSCLC patients | gefitinib, erlotinib, afatinib | RCT(16) Non-RCT(0) | 2962 | Rash，diarrhea，elevated liver transaminase | |  |
| Burotto[^2^](#_ENREF_2) | 2015 | Metastatic NSCLC patients | gefitinib, erlotinib | RCT(28) Non-RCT(0) | 11270 | Pruritus, rash acne, anorexia, diarrhea, nausea, fatigue, paronychia, anemia, dry skin, vomiting, transaminitis, interstitial lung disease | |  |
| Ding[^3^](#_ENREF_3) | 2013 | Advanced NSCLC patients | gefitinib, erlotinib, afatinib | RCT(16) Non-RCT(0) | 2535 | Diarrhea, elevated liver enzymes, rash, stomatitis, fatigue, nausea, paronychia, pruritus, vomiting , appetite loss, pneumonitis | |  |
| Hong[^4^](#_ENREF_4) | 2016 | Advanced NSCLC patients | gefitinib | RCT(23) Non-RCT(0) | 9054 | Interstitial lung disease, pneumonitis, hemoptysis | |  |
| Lin[^5^](#_ENREF_5) | 2018 | EGFR-positive NSCLC patients | gefitinib, erlotinib, afatinib, dacomitinib, osimertinib | RCT(11) Non-RCT(0) | 3145 | Skin rash, elevated alanine aminotransferase/aspartate aminotransferase, diarrhea, acne, mucositis, nails change | |  |
| Suh[^6^](#_ENREF_6) | 2018 | Advanced NSCLC patients | gefitinib, erlotinib, afatinib, osimertinib | RCT(0) Non-RCT(253) | 15713 | Pneumonitis | |  |
| Yang[^7^](#_ENREF_7) | 2017 | NSCLC patients | gefitinib, erlotinib, afatinib | RCT(8) Non-RCT(82) | 17621 | Rash, diarrhea, liver dysfunction, nausea, vomiting, paronychia, asthenic conditions, oral ulcer, pruritus desquamation, eye change, stomatitis, constipation, interstitial lung disease, neutropenia | |  |
| Zhang[^8^](#_ENREF_8) | 2017 | Advanced EGFR-positive NSCLC patients after failure of first-generation tyrosine kinase inhibitors | afatinib | RCT(0) Non-RCT(5) | 545 | Rash, ance, diarrhoea | |  |
| Zhang[^9^](#_ENREF_9) | 2018 | Advanced NSCLC patients | gefitinib, erlotinib | RCT(3) Non-RCT(28) | 8054 | Skin rash, diarrhea, nausea, vomiting, fatigue, anorexia, interstitial lung disease, stomatitis, elevated liver enzymes, infection, neutropenia | |  |

EGFR-TKI: epidermal growth factor receptor-tyrosine kinase inhibitor

NSCLC: non-small cell lung cancer

RCT: randomized controlled trial

Reference

1 Zhang, Y. *et al.* Optimized selection of three major EGFR-TKIs in advanced EGFR-positive non-small cell lung cancer: a network metaanalysis. *Oncotarget* **7**, 20093-20108 (2016).

2 Mauricio, B., Manasanch, E. E., Julia, W. & Tito, F. Gefitinib and erlotinib in metastatic non-small cell lung cancer: a meta-analysis of toxicity and efficacy of randomized clinical trials. *Oncologist* **20**, 400-410 (2015).

3 Ding, P. N. *et al.* Risk of treatment-related toxicities from EGFR tyrosine kinase inhibitors: a meta-analysis of clinical trials of gefitinib, erlotinib, and afatinib in advanced EGFR-mutated non–small cell lung cancer. *Journal of Thoracic Oncology* **12**, 633-643 (2017).

4 Hong, D., Zhang, G., Zhang, X. & Lian, X. Pulmonary toxicities of gefitinib in patients with advanced non-small-cell lung cancer: a meta-analysis of randomized controlled trials. *Medicine* **95** (2016).

5 Lin, J.-Z., Ma, S.-K., Wu, S.-X., Yu, S.-H. & Li, X.-Y. A network meta-analysis of nonsmall-cell lung cancer patients with an activating EGFR mutation: Should osimertinib be the first-line treatment? *Medicine* **97** (2018).

6 Suh, C. H. *et al.* Pneumonitis in advanced non-small-cell lung cancer patients treated with EGFR tyrosine kinase inhibitor: Meta-analysis of 153 cohorts with 15,713 patients: Meta-analysis of incidence and risk factors of EGFR-TKI pneumonitis in NSCLC. *Lung Cancer* **123**, 60-69 (2018).

7 Yang, Z. *et al.* Comparison of gefitinib, erlotinib and afatinib in non‐small cell lung cancer: A meta‐analysis. *International journal of cancer* **140**, 2805-2819 (2017).

8 Zhang, Y. *et al.* The efficacy and toxicity of afatinib in advanced EGFR-positive non-small-cell lung cancer patients after failure of first-generation tyrosine kinase inhibitors: a systematic review and meta-analysis. *Journal of thoracic disease* **9**, 1980 (2017).

9 Zhang, W., Wei, Y., Yu, D., Xu, J. & Peng, J. Gefitinib provides similar effectiveness and improved safety than erlotinib for east Asian populations with advanced non–small cell lung cancer: a meta-analysis. *BMC cancer* **18**, 780 (2018).
